# Effects of the PLK4 inhibitor Centrinone on the biological behaviors of acute myeloid leukemia cell lines

**DOI:** 10.3389/fgene.2022.898474

**Published:** 2022-08-16

**Authors:** Xing-Ru Mu, Meng-Meng Ma, Zi-Yi Lu, Jun Liu, Yu-Tong Xue, Jiang Cao, Ling-Yu Zeng, Feng Li, Kai-Lin Xu, Qing-Yun Wu

**Affiliations:** ^1^ Blood Diseases Institute, Xuzhou Medical University, Xuzhou, Jiangsu, China; ^2^ Department of Hematology, the Affiliated Hospital of Xuzhou Medical University, Xuzhou, Jiangsu, China; ^3^ Department of Cell Biology and Neurobiology, Xuzhou Medical University, Xuzhou, China

**Keywords:** acute myeloid leukemia, PLK4, small molecule inhibitors, centrinone, biological behaviors

## Abstract

Polo-like kinase 4 (PLK4), a key regulator of centriole biogenesis, is frequently overexpressed in cancer cells. However, roles and the mechanism of PLK4 in the leukemiagenesis of acute myeloid leukemia (AML) remain unclear. In this study, the PLK4 inhibitor Centrinone and the shRNA knockdown were used to investigate roles and the mechanism of PLK4 in the leukemiagenesis of AML. Our results indicated that Centrinone inhibited the proliferation of AML cells in a dose- and time-dependent manner *via* reduced the expression of PLK4 both in the protein and mRNA levels. Moreover, colony formation assay revealed that Centrinone reduced the number and the size of the AML colonies. Centrinone induced AML cell apoptosis by increasing the activation of Caspase-3/poly ADP-ribose polymerase (PARP). Notably, Centrinone caused the G2/M phase cell cycle arrest by decreasing the expression of cell cycle-related proteins such as Cyclin A2, Cyclin B1, and Cyclin-dependent kinase 1 (CDK1). Consistent with above results, knockdown the expression of PLK4 also inhibited cell proliferation and colony formation, induced cell apoptosis, and caused G2/M phase cell cycle arrest without affecting cell differentiation. All in all, this study suggested that PLK4 inhibited the progression of AML *in vitro*, and these results herein may provide clues in roles of PLK4 in the leukemiagenesis of AML.

## Introduction

Acute myeloid leukemia (AML) is a malignant proliferative disease of bone marrow hematopoietic stem cells with a high mortality rate (Siegel et al., 2020). Continuing for nearly four decades, the treatment of AML is still dominated by induction chemotherapy combined with allogeneic hematopoietic stem cell transplantation, with low cure rates and certain limitations (Vakiti and Mewawalla. 2021). AML patients often have genetic abnormalities which were associated with the poor prognosis. Targeted therapy for AML with the therapeutic advantages of selective removal of leukemia cells and the ability to overcome drug resistance has received increasing attention in recent years (Kayser and Levis. 2018). Although many inhibitors were used in the clinical trials, the therapeutic effect was still unsatisfactory. Thus, it is necessary to explore new therapeutic strategies for AML, particularly individualized molecularly targeted therapies.

Polo-like kinases (PLKs) are a family of serine/threonine kinases, which are involved in the centrosome replication and cell cycle regulation (Archambault and Glover. 2009; de Carcer et al., 2011). PLK family members feature similar structures with an N-terminal kinase catalytic domain and C-terminal Polo-box structural domains (PBDs) (Strebhardt. 2010; Archambault et al., 2015). PLK4, also known as serine/threonine kinase (SAK), contrasts with other PLKs in that it contains only a PBD structural domain (Leung et al., 2002; Sillibourne and Bornens. 2010). PLK4 high expression contributes to excessive centriole formation, which causes genomic instability and tumorigenesis (Holland et al., 2010).

The aberrant expression of PLK4 has a close relationship with a variety of malignancies. Although many studies have been done on roles of PLK4, there was a controversy in whether PLK4 promoted or suppressed the progression of cancers. A series of studies indicated that PLK4 promoted the progression of cancers. PLK4 was prevailing high expressed in the breast cancer, with only 2.6% samples being negative (Li et al., 2016). Similarly, over half of the gastric cancer cell lines showed significantly elevated PLK4 mRNA levels (Shinmura et al., 2014). Kawakami et al. also demonstrated that the PLK4 was high expressed in lung adenocarcinoma tissues compared to normal lung tissues which indicated a low overall survival and progression-free survival (Kawakami et al., 2018a). Besides, PLK4 was also overexpressed and promoted the progression of melanoma, hepatocellular carcinoma and brain tumors (Denu et al., 2018). Similar to roles of PLK4 in the solid tumors, in 80% of classical Hodgkin’s lymphoma were high expressed and promoted its progression (Ward et al., 2015). But the expression of PLK4 was decreased in some hematologic malignancies compared to normal tissues, such as in 82.0% of lymphomas, 80.5% of myelodysplastic syndromes (MDS) and 60% of acute lymphoblastic leukemia (ALL) and did not display oncogene roles. In our previous study, the RNA-Seq analysis revealed that PLK4 was highly expressed in AML and had a close relationship with the overall survival of AML patients. However, roles and mechanisms of PLK4 in the leukemiagenesis of AML were still unclear. In this study, effects of PLK4 inhibitor Centrinone and lentivirus-mediated PLK4 knockdown on the biological behaviors of AML cell lines were used to investigate roles and mechanisms of PLK4 in the pathology of AML.

In recent years, due to key roles of PLK4 in the progression of cancers, several PLK4 inhibitors have been identified, but the clinical therapeutic effects on cancers were still unsatisfactory (Holland and Cleveland. 2014; Mason et al., 2014; Liu et al., 2017; Lei et al., 2018). Thus, to elucidate roles and mechanisms of PLK4 in the progression of cancers was important for the new drug design and screen. In this study, effects of PLK4 inhibitor Centrinone and lentivirus mediated knockdown the expression of PLK4 on the AML cell lines’ biological behaviors such as cell proliferation, apoptosis, cell cycle, and colony formation were investigated to clarify roles and mechanisms of PLK4 in the leukemiagenesis of AML. Our results suggested that PLK4 inhibited AML cell proliferation, colony formation, induced AML cell apoptosis and caused the G2/M cell cycle arrest by affecting the activation of Caspase-3/PARP and the expression of cell cycle-related proteins such as Cyclin A2, Cyclin B1 and CDK1. Thus, these results herein may provide clues in roles and mechanisms of PLK4 in the leukemiagenesis of AML.

## Materials and methods

### Cell culture and reagents

Human AML cell lines MOLM-13, KG-1 and OCI-AML3 were cultured in RPMI-1640/IMDM (HyClone, United States) medium, supplemented with 10% fetal bovine serum (FBS; Gibco, United States) and 1% penicillin/streptomycin mixture. All cells were incubated at 37°C with 5% CO_2_ in a humidified atmosphere. Centrinone (MCE, China) was dissolved in the dimethyl sulfoxide (DMSO; Sigma, United States) to a storage concentration of 50 mM.

### Cell viability assays

AML cells were seeded into a 96-well plate at a density of 20,000 cells/well for cell viability assay. Cells were treated with different Centrinone concentrations or the vehicle DMSO for 24–96 h, then 10 μL/well Cell Counting Kit-8 (CCK-8; Absin, China) was added to the medium and incubated at 37°C for 3 h, followed by measuring absorbance at 450 nm by Spectra Max M2 Microplate Reader (Molecular Devices, United States).

### Cell apoptosis assay

AML cells with the designed treatment were collected and incubated with Annexin V-APC and 7-Aminoactinomycin D (7-AAD; BD, United States) for 15 min. Then, cells were subjected to flow cytometry. The rates of apoptotic cells were acquired on the Navios flow cytometer (Beckman Coulter, United States).

### Cell cycle assay

AML cells were treated with a serum-free medium to synchronize cells. Then cells were treated with Centrinone at different concentrations for 48 h. Cells were collected and fixed with 70% ethanol overnight at 4°C, then stained with propidium iodide (PI)/RNase staining buffer (BD, United States) for 15 min. The DNA content was monitored by the Navios flow cytometer (Beckman Coulter, United States), and the data was analyzed using FlowJo Version 7.6 software (TreeStar, United States).

### Cell differentiation assay

AML cells were treated with different concentrations of Centrinone and incubated with fluorescein isothiocyanate (FITC) CD 11b (BD, United States) for 30 min. Then cells were subjected to flow cytometry and acquired on Navios flow cytometer (Beckman Coulter, United States).

### colony formation assay

AML cells (300 cells/well) were inoculated in the 6-well plates filled with methylcellulose, treated with different concentrations of Centrinone, and incubated in an incubator at 37°C, 5% CO_2_ for 2 weeks. Colonies were observed and counted with an inverted microscope, stained with Giemsa (Beyotime, China), and photographed.

### RNA Isolation and quantitative real-time PCR

Total RNA was extracted using Trizol reagent (Invitrogen, United States), and cDNA was synthesized by reverse transcription with OligodT as a primer and M-MLV reverse transcriptase (Invitrogen, United States). PLK4 and GAPDH were amplified by real-time PCR on the Light Cycler480 II system (Roche, United States) using Platinum SYBR Green qPCR Super Mix-UDG kit (Roche, United States), and the relative expression levels of each group of genes were calculated by relative gene quantification (2^-∆∆Ct^) using GAPDH expression levels as an internal reference. The primer sequences were as follows:

PLK4 forward: 5′-GTG​GGG​AAA​TCA​AGA​AAC​CA-3′;

PLK4 reverse: 5′-GGT​GGC​TCC​ATA​CCC​CTA​GT-3′;

GADPH forward: 5′-CGA​GAT​CCC​TCC​AAA​ATC​AA-3′;

GADPH reverse: 5′-TGT​GGT​CAT​GAG​TCC​TTC​CA-3′.

### western blot analysis

Total protein was extracted using radio immunoprecipitation assay (RIPA) lysis buffer (Beyotime, China) containing protease inhibitor cocktail tablets (KeyGen, China) and phosphatase inhibitor cocktail (KeyGen, China), according to operating instructions. Protein concentrations were measured using a bicinchoninic acid (BCA) assay (Beyotime, China), and then equal amounts of total protein were separated by electrophoresis on a 10% polyacrylamide gel (Bio-Rad, United States). The protein extracts were transferred onto polyvinylidene fluoride (PVDF) membranes (Millipore, Billerica, United States), and then membranes were blocked in Tris-buffered saline (contained 0.05% Tween) with 5% defatted milk for 1.5 h. Then membranes were incubated with primary antibodies at 4°C overnight. The primary antibodies were used with a concentration of 1: 1,000 as follows: PLK4 (Proteintech, China), Caspase-3, PARP, Cyclin A2, Cyclin B1, CDK1, signal transducers and activators of transcription 3 (STAT3), and p-STAT3 (Cell Signaling Technology, United States). GAPDH (Bioworld, United States) was used as the endogenous control. Goat anti-rabbit or goat anti-mouse secondary antibodies labeled with horseradish peroxidase (HRP) (1: 5,000, Bioworld, United States) were hybrid bindings at room temperature for 1.5 h, and signals were detected with the AI600 Imaging System (General Electric, United States) using an enhanced chemiluminescence (ECL) kit (Bio-Rad, United States).

### Construction of lentiviral interference vector and transfection

The specific shRNA sequence of the *PLK4* was annealed and ligated with the pLV-shRNA-EGFP linear vector after double digestion with *EcoR*I and *BamH*I to construct the recombinant interfering vector OCI-AML3-sh-PLK4-1/2, which was sent to Invitrogen for sequencing. The virus particles were packaged using a triple plasmid system, and ultracentrifugation was used to concentrate the virus and stored at −80°C. 1 day before transfection, OCI-AML3 cells were seeded into 24-well plates at a concentration of 5 × 10^4^ cells per well. Appropriate doses of lentivirus and transfection enhancers were co-cultured with cells. Both empty vector and PLK4 knockdown lentivirus vectors expressed green fluorescent protein (GFP) and puromycin resistance genes. 2 days later, 8 μg/ml puromycin (VICMED, China) was added to the culture medium to screen stably transfected cells. Infection efficiency was determined by GFP and then validated by RT-PCR and Western blot.

### Statistical analysis

All experiments were repeated at least three times, and the data were statistically analyzed and processed with SPSS16.0 software or GraphPad Prism7.03 software. The measurement data were expressed as mean ± standard deviation, and *t*-test was used for comparison of two sample means, and one-way ANOVA was used for comparison of more than two sample means. The test level was *α* = 0.05, and *p < 0.05 indicated that the difference was statistically significant.

## Results

### Centrinone inhibited the proliferation of AML cells in a dose- and time-dependent manner

**FIGURE 1:**
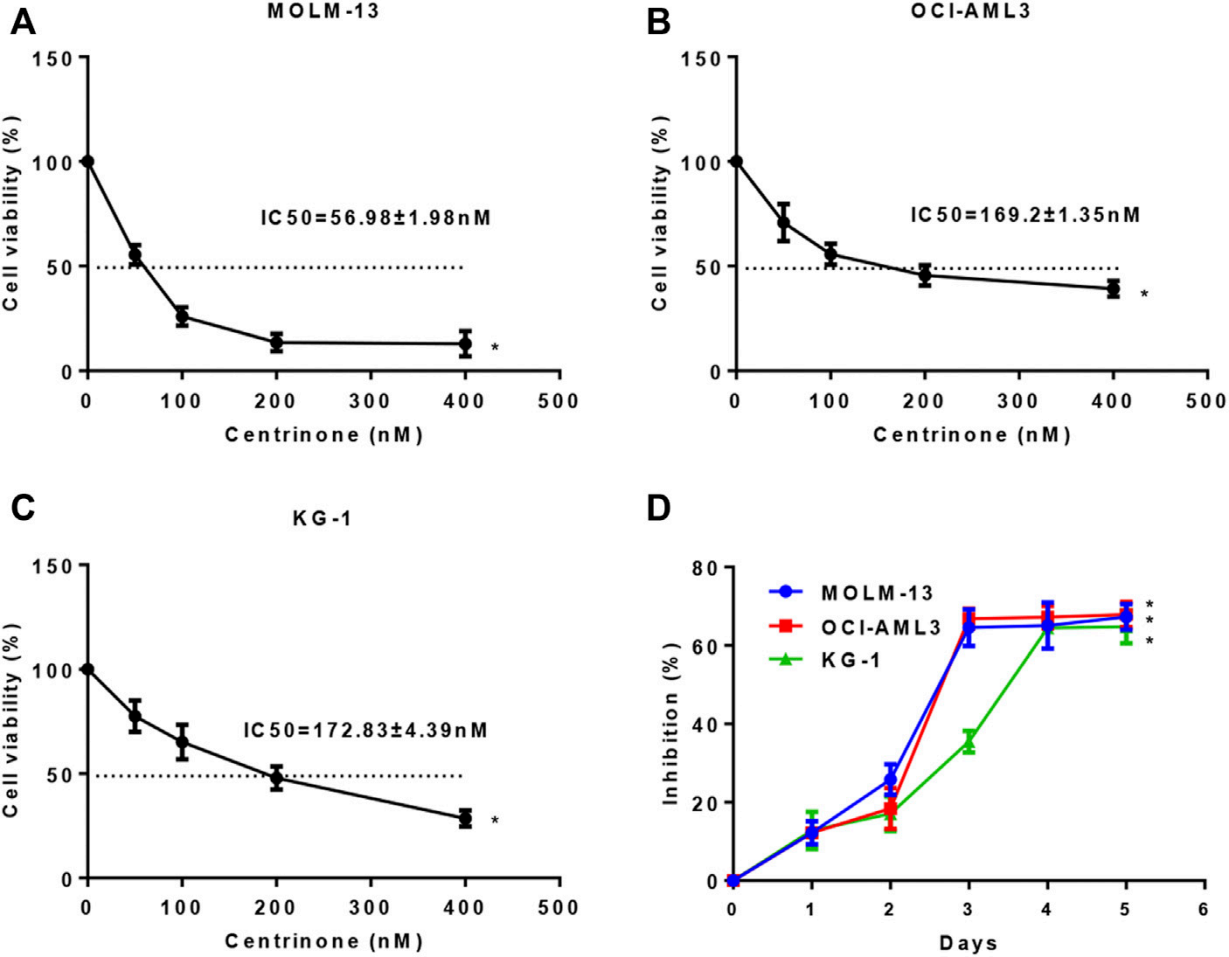
Centrinone inhibited the proliferation of AML cells in a dose- and time-dependent manner. **(A–C)** Effects of different Centrinone concentrations (0, 50, 100, 200, 400 nM) on the proliferation of AML cells were determined by treatment MOLM-13, OCI-AML3, and KG-1 cells for 72 h, then CCK-8 was added, incubated for 3 h, finally the OD450 absorbance was detected. **(D)** The indicated Centrinone concentrations was used to treat MOLM-13 (54.26 nM), OCI-AML3 (177.7 nM), and KG-1 (189.9 nM) cells for 1-5 days to detect the effect of Centrinone on the proliferation of AML cells at different treatment times. Columns, means (*n* ≥ 3); bars, *SD*. *p < 0.05 versus the control group.

In order to investigate effects of Centrinone on the proliferation of MOLM-13, OCI-AML3, and KG-1 AML cell lines, CCK-8 assay was done. AML cells were inoculated in 96-well plates at a density of 2×10^4^ cells/well, and different Centrinone concentrations (50, 100, 200 and 400 nM) were used to treat AML cells for 24, 48 and 72 h. Our results indicated that the proliferation of OCI-AML3, and KG-1 AML cells gradually decreased with Centrinone concentrations increased compared with the control group (Figures 1A–C), while the proliferation of MOLM-13 gradually decreased when the Centrinone concentrations were between 50–200 nM, and no obvious changes were observed when the Centrinone concentrations between 200–400 nM which might be caused by the high drug concentrations (Figures 1A–C). Similarly, the proliferation of MOLM-13, OCI-AML3, and KG-1 AML cells gradually decreased with the Centrinone treatment time increased compared with the control group (Figure 1D). These results suggested that Centrinone inhibited the proliferation of AML cell lines in a dose- and time-dependent manner.

### Centrinone inhibited the expression of PLK4 in AML Cell lines

**FIGURE 2:**
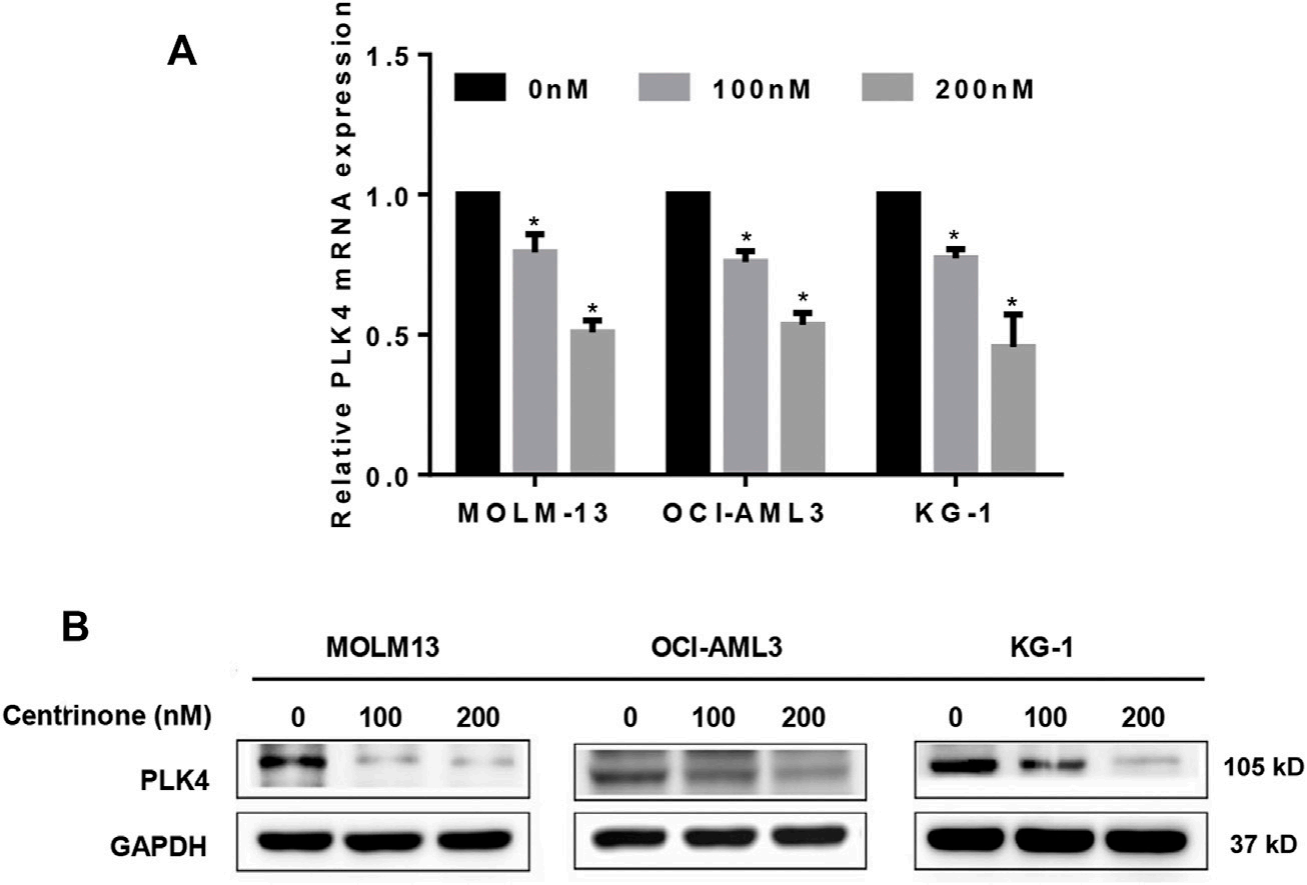
Centrinone inhibited the expression of PLK4. **(A)** The mRNA expression levels of PLK4 were detected by RT-PCR after AML cells treated with different concentrations of Centrinone for 72 h. Compared with the control group, *p < 0.05. **(B)** The expression of PLK4 were detected by Western blot in AML cells treated with diverse concentrations of Centrinone for 72 h and the expression of GAPDH was used as an internal control.

In order to elucidate effects of Centrinone concentrations (0, 100, 200 nM) on the expression of PLK4 in the MOLM-13, OCI-AML3, and KG-1 cells, the RT-PCR and Western blot experiments were done. Our results indicated that Centrinone inhibited the expression of PLK4 both in the mRNA and protein levels with the increased Centrinone concentrations in the MOLM-13, OCI-AML3 and KG-1 cells (Figures 2A,B).

### Centrinone induced the apoptosis of AML cells

**FIGURE 3:**
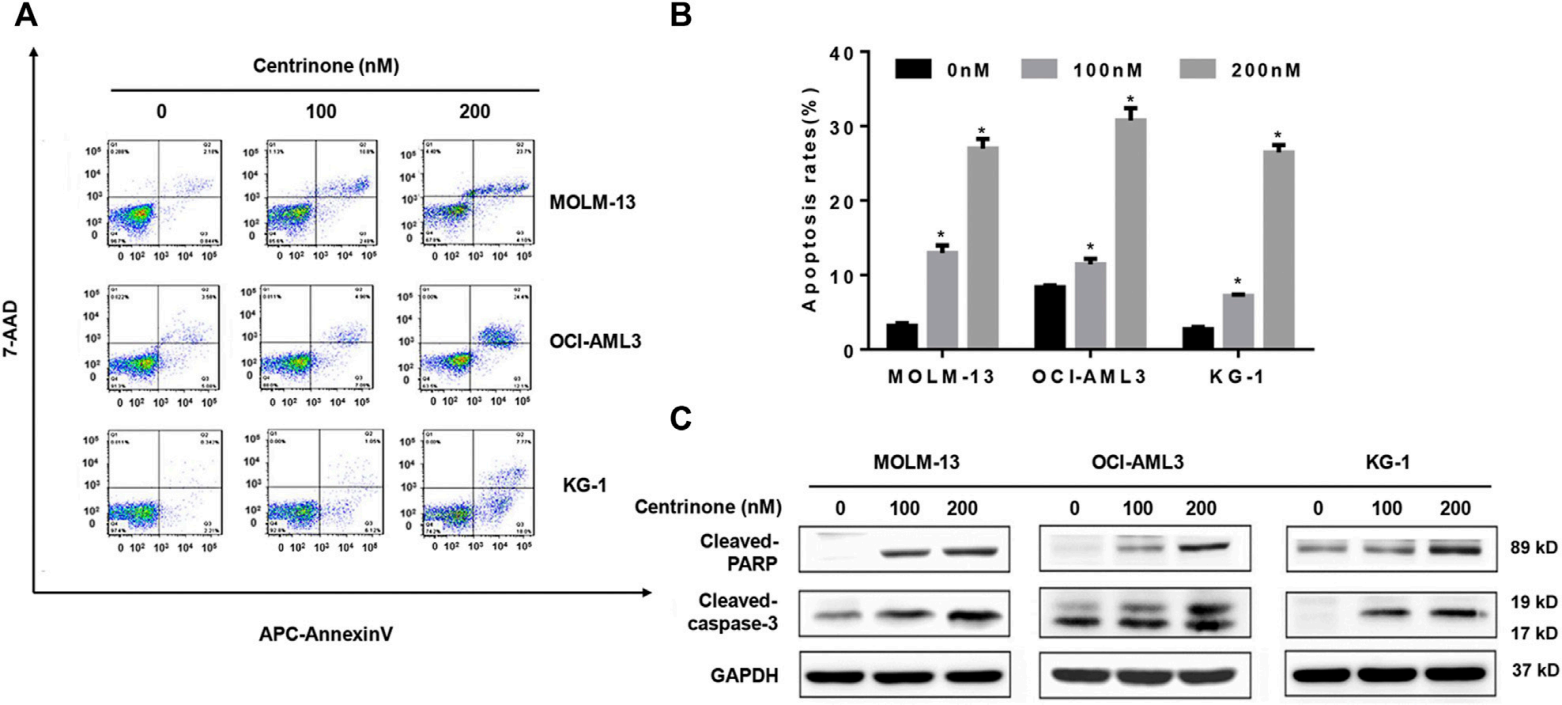
Centrinone induced the apoptosis of AML cells *via* increasing the expression of cleaved Caspase-3 and cleaved PARP. **(A)** The apoptosis of AML cells treated with different Centrinone concentrations after 72 h was analyzed by flow cytometry with Annexin V/7-AAD double staining. **(B)** Statistical analysis of apoptosis of AML cells treated by Centrinone (compared with the control group, *p < 0.05). **(C)** The expression of cleaved Caspase-3 and cleaved PARP were detected by Western blot.

In order to investigate effects of Centrinone on the apoptosis of AML cells, flow cytometry was used to detect the number of double staining cells with Annexin V and 7-AAD. The early apoptotic cells were defined as Annexin V^+^/7-AAD^-^, late apoptotic cells were defined as Annexin V^+^/7-AAD^+^ and the total percentage of apoptotic cells was the sum of early apoptotic cells and late apoptotic cells. Our results suggested that the percentage of apoptotic cells in all three AML cell lines increased with the elevated Centrinone concentrations (0, 100, 200 nM) after 72 h treatment (Figures 3A,B). The expression of apoptosis-related proteins was also detected to clarify the mechanism of Centrinone led to the AML cell apoptosis. As shown in Figure 3C, the expression of cleaved Caspase-3 and cleaved PARP were significantly increased in AML cells after Centrinone treatment. Thus, these observations suggested that Centrinone induced the apoptosis of AML cells *via* the activation of Caspase-3 and PARP pathway.

### Centrinone induced AML cells in the G2/M phase cell cycle arrest

**FIGURE 4:**
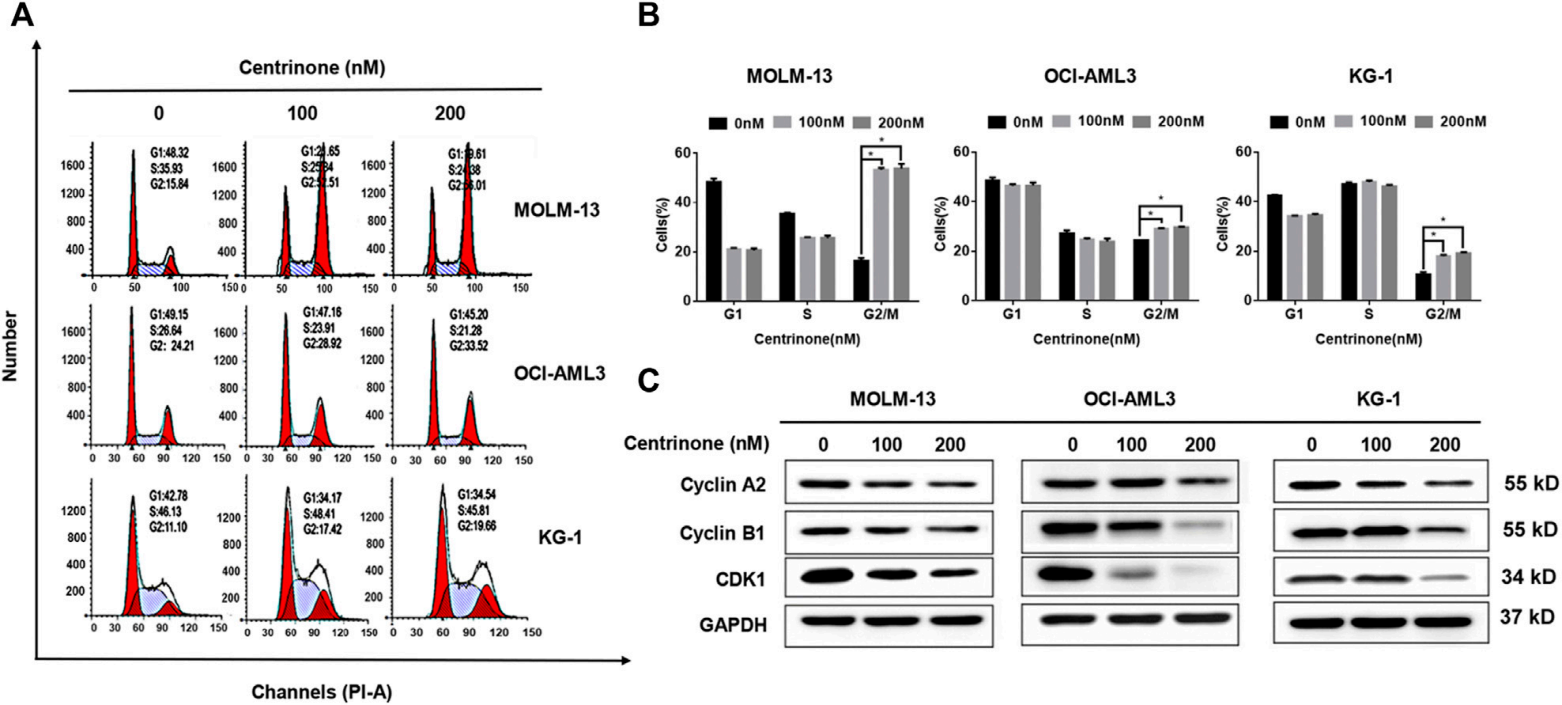
Centrinone caused AML cells in the G2/M phase cell cycle arrest by reducing the expression of Cyclin A2, Cyclin B1, and CDK1. **(A)** AML cells were treated by Centrinone for 48 h, and the cell cycle of ethanol-fixed cells with PI staining was analyzed by flow cytometry. **(B)** Statistical analysis of the distribution of G1, S and G2/M phase AML cells treated by Centrinone (compared with the control group, *p < 0.05). **(C)** The expression of Cyclin A2, Cyclin B1, and CDK1 were detected by Western blot.

In order to clarify effects of the Centrinone on the cell cycle of AML cell lines, the PI stain experiments were done. MOLM-13, OCI-AML3, and KG-1 cells were treated with different Centrinone concentrations (0, 100, 200 nM) for 48 h. DNA from ethanol-fixed cells was stained with PI, and the DNA content was measured by flow cytometry. The number of cells in the G2/M phase increased continuously with Centrinone concentrations increased (Figures 4A,B). At the same time, the expressions of cell cycle-related proteins were detected to explore the mechanism of Centrinone caused the cell cycle arrest of AML cells. As shown in Figure 4C, the expression of Cyclin A2, Cyclin B1 and CDK1 were significantly reduced in AML cells after Centrinone treatment. This result suggested that Centrinone arrested the cell cycle in the G2/M phase by reducing the expression of Cyclin A2, Cyclin B1, and CDK1.

### Centrinone suppressed the colony formation of AML cells

**FIGURE 5:**
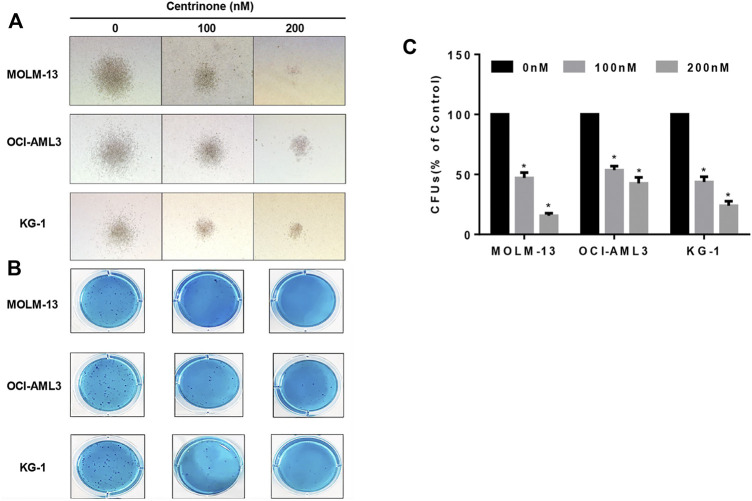
Centrinone reduced the clonogenic ability of AML cell lines. AML cells were co-incubated with Centrinone for 14 days. Single colonies were observed and counted under the microscope. **(A)** Microscopic observation of cell colony generation size (100×). **(B)** The number of colony generations was observed after Giemsa staining. **(C)** Statistical analysis of the number of colony formation of MOLM-13, OCI-AML3, and KG-1 AML cells (Compared with the control group, *p < 0.05).

The colony formation assay was conducted to evaluate effects of Centrinone on colony formation ability of AML cell lines. MOLM-13, OCI-AML3, and KG-1 cells were seeded in methylcellulose medium and treated with different Centrinone concentrations (0, 100, and 200 nM) for 14 days, and the number of colonies was counted under the microscope. Compared with the control group, the quantity and size of cell colonies markedly decreased and significantly reduced after Centrinone treatment for 14 days (Figures 5A–C).

### Centrinone had no effect on the differentiation of AML cells

**FIGURE 6:**
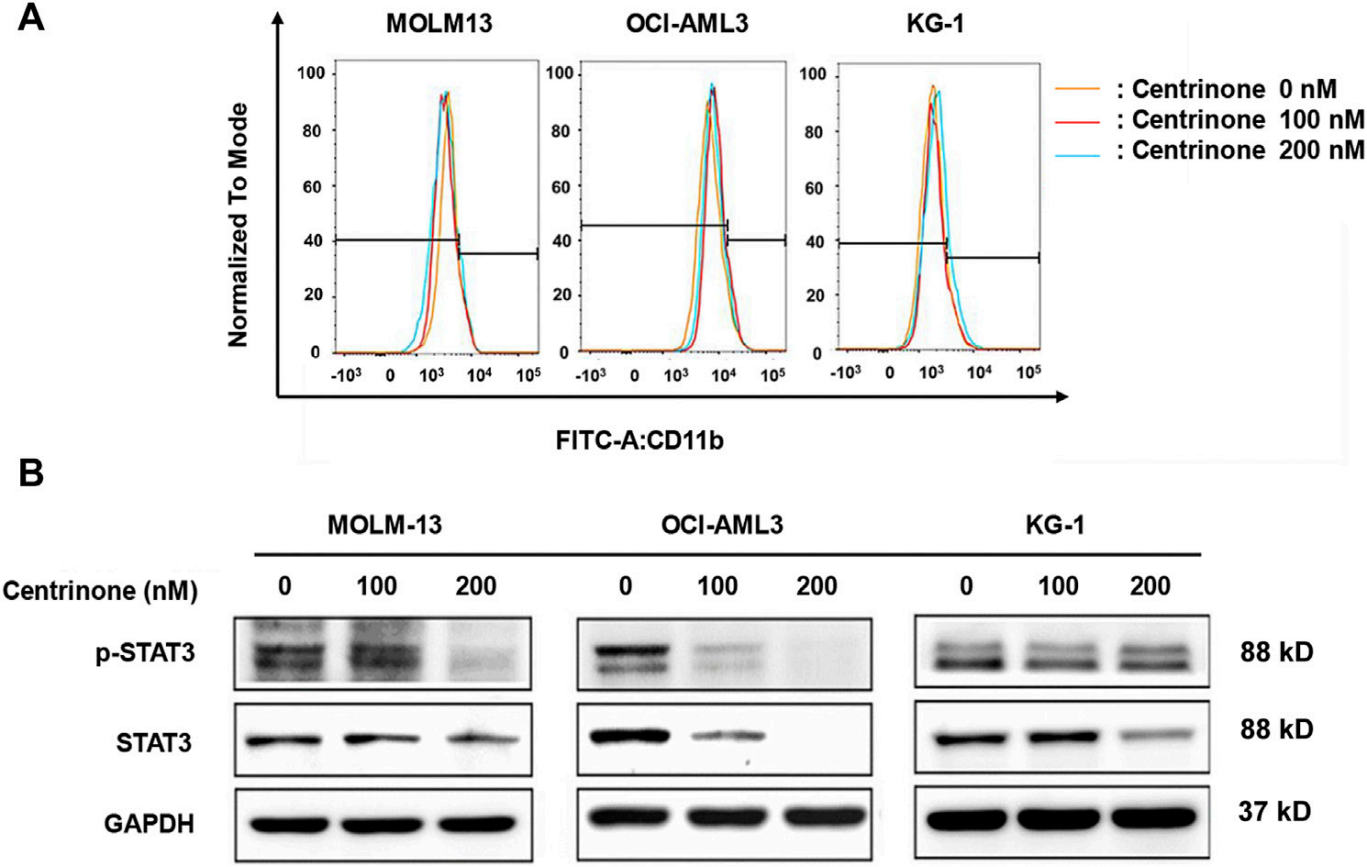
Effects of Centrinone on the expression of CD11b and the activation of STAT3 in AML cell lines. **(A)** Effects of Centrinone on the expression of myeloid cell differentiation marker CD11b in AML cell lines. AML cells were treated with Centrinone for 48 h, and the expression of CD11b was analyzed by flow cytometry. **(B)** Centrinone inhibited the activation of STAT3. AML cells treated with different concentrations of Centrinone for 72 h, and then the expression of STAT3 and p-STAT3 were detected by Western blot. The expression of GAPDH was used as an internal control.

The blockage of cell differentiation was one reason for the pathology of AML (Denu et al., 2018). In order to elucidate whether Centrinone affected the differentiation of AML cell lines, the expression of myeloid differentiation maker CD 11b was determined. MOLM-13, OCI-AML3, and KG-1 cells were treated with different Centrinone concentrations (0, 100, 200 nM) for 48 h. Then, cells were collected, stained with FITC CD11b, and detected by flow cytometry. No significant cell differentiation changes were observed compared with the control group (Figure 6A).

### Centrinone inhibited the activation of STAT3 in AML cells

Previous study indicated that STAT3 was involved in the regulation of centrosome clustering in cancer cells (Morris et al., 2017). In order to investigate whether Centrinone affected the activation of STAT3, the expression of STAT3 and p-STAT3 in AML cells was detected by Western blot. MOLM-13, OCI-AML3, KG-1 cells were treated with different Centrinone concentrations (0, 100, 200 nM) for 72 h, and then cell harvest, protein extraction and the Western blot experiment was done. As shown in Figure 6B, the expression of STAT3 and p-STAT3 significantly decreased with the Centrinone concentrations increased, which suggested that PLK4 inhibited the progression of AML *via* reducing the activation of STAT3.

### Knockdown the expression of PLK4 inhibited the Cell proliferation and suppressed the clonogenic ability of OCI-AML3 cells

**FIGURE 7:**
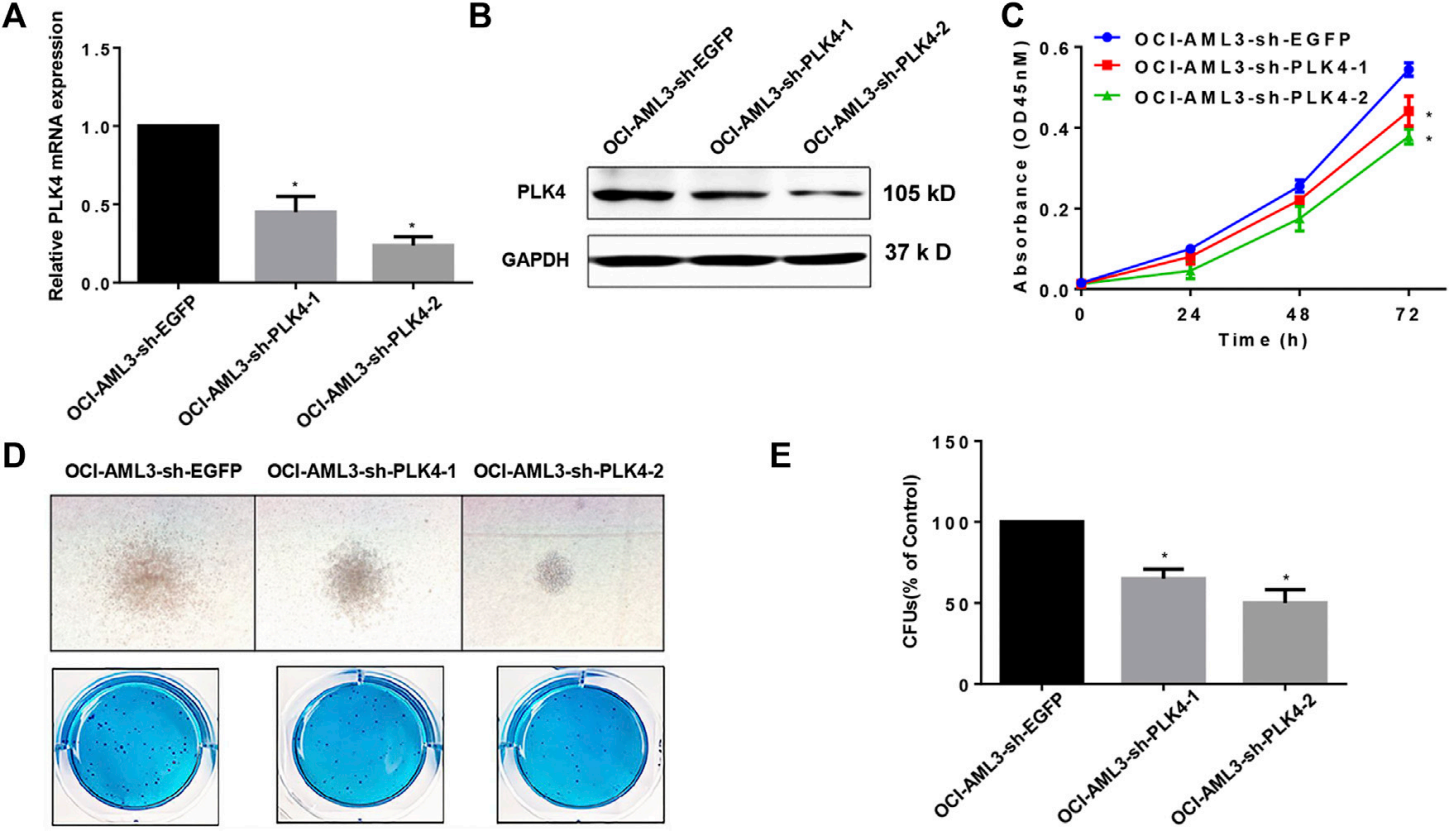
Knockdown the expression of PLK4 inhibited the proliferation and the colony formation of OCI-AML3 cells. **(A)** Relative mRNA expression levels of PLK4 in the OCI-AML3 cells after lentivirus medicated transduction, *p < 0.05, compared with OCI-AML3-sh-EGFP group. **(B)** Expression of PLK4 in transfected OCI-AML3 cells was detected by Western blot. **(C)** Knockdown the expression of PLK4 inhibited the proliferation of OCI-AML3 cells. *p < 0.05, compared with OCI-AML3-sh-EGFP group. **(D)** Knockdown the expression of PLK4 inhibited the size and the numbers of the colony formation. Microscopic observation of cell colony generation size (100×) and the number of colony generations was observed after Giemsa staining. **(E)** Statistical analysis of the number of cell-forming colonies (compared with control, *p < 0.05).

The OCI-AML3 cells that stably knockdown the expression of PLK4 were obtained by the lentivirus-mediated transduction and sorted by the flow cytometry. The expression of PLK4 was significantly decreased both in the mRNA and protein levels in the knockdown groups compared with the control group (Figures 7A,B). In order to investigate effects of knockdown the expression of PLK4 on the proliferation of OCI-AML3 cells, CCK-8 assay was done. OCI-AML3-sh-EGFP, OCI-AML3-sh-PLK4-1 and OCI-AML3-sh-PLK4-2 cells were inoculated uniformly in 96-well plates at a density of 2 × 10^4^ cells/well, at 24, 48 and 72 h, CCK-8 was added and incubated for 3 h, and then the OD450 was detection. Similar to results of Centrinone inhibition experiments, knockdown the expression of PLK4 significantly suppressed the proliferation of OCI-AML3 cells compared with the control group (Figure 7C). At the same time, effects of knockdown the expression of PLK4 on the clonogenic ability of OCI-AML3 cells were investigated by colony formation assay. As shown in Figures 7D,E, knockdown the expression of PLK4 reduced the number and the size of colonies compared to the control group.

### Knockdown the expression of PLK4 promoted the apoptosis, arrested Cell cycle, while had no effect on the differentiation of OCI-AML3 cells

**FIGURE 8:**
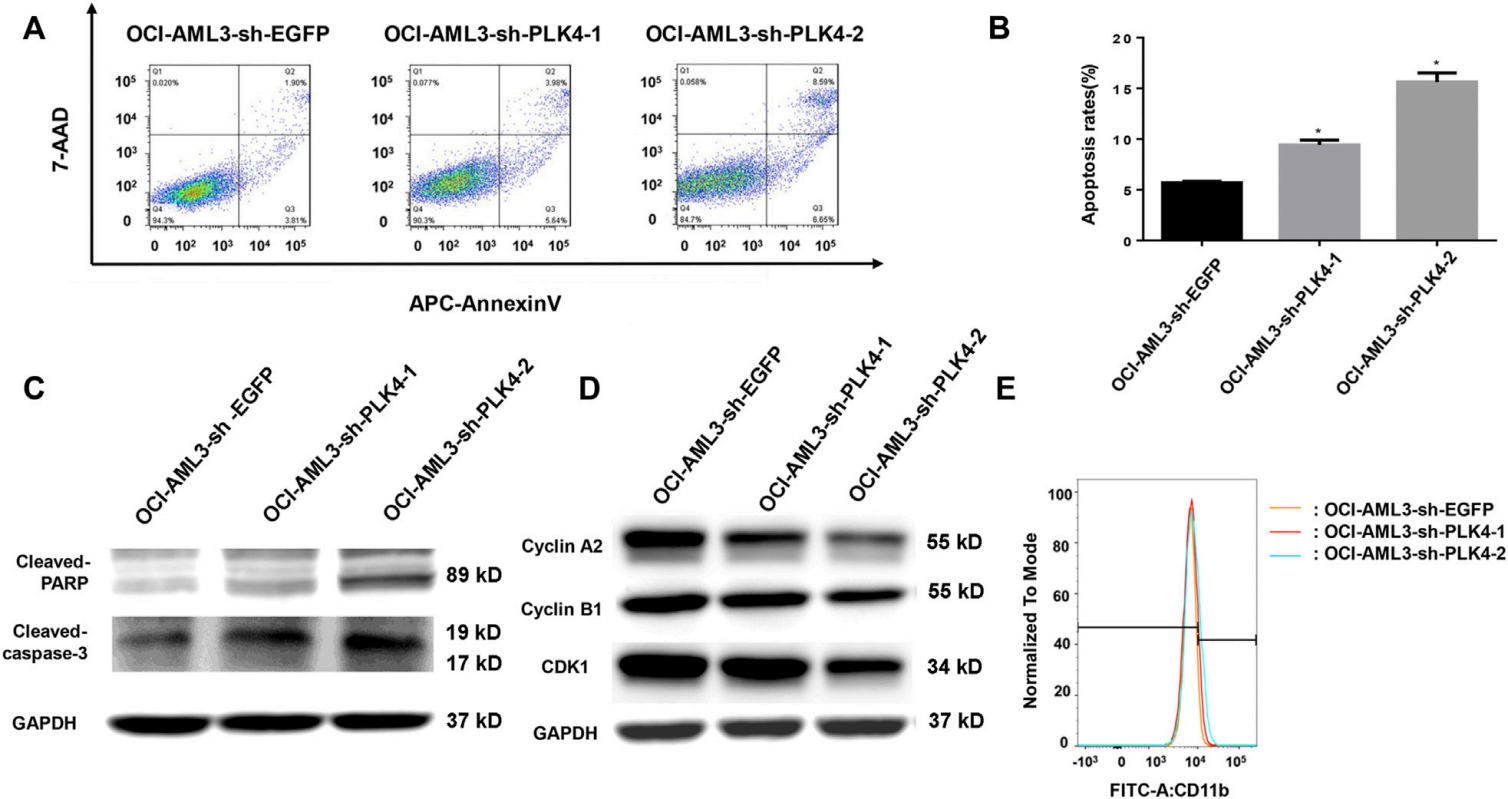
Knockdown the expression of PLK4 induced the apoptosis and the cell cycle arrest of OCI-AML3, while had no effect on the differentiation of OCI-AML3 cells. **(A)** The apoptosis of each group was analyzed by flow cytometry with Annexin V/7-AAD double stain. **(B)** Statistical analysis of the apoptosis of PLK4 knockdown OCI-AML3 cells (compared with the control group, *p < 0.05). **(C)** The expression of cleaved Caspase-3 and cleaved PARP was detected by Western blot. **(D)** The expression of Cyclin A2, Cyclin B1 and CDK1 was detected by Western blot. **(E)** Effect of PLK4 on the differentiation of OCI-AML3 cells were stained with FITC CD11b and analyzed by flow cytometry.

The blockage of cell apoptosis and cell differentiation were important for the leukemiagenesis of AML. In order to detect effects of knockdown the expression of PLK4 on cell apoptosis and cell differentiation, the cell numbers with Annexin V and 7-AAD double stain, the expression of apoptosis related proteins and the myeloid differentiation maker CD11b was determined by flow cytometry and Western blot. As shown in Figures 8A,B, the proportion of apoptotic cells was significantly higher in the PLK4 knockdown groups than that of the control group. At the same time, knockdown the expression of PLK4 promoted the expression of cleaved Caspase-3 and cleaved PARP (Figure 8C). Similar to the result of Centrinone inhibition, knockdown the expression of PLK4 significantly reduced the expression of cell cycle-related proteins Cyclin A2, Cyclin B1 and CDK1 (Figure 8D), which indicated the cell cycle arrested. No significant cell differentiation changes were observed between the PLK4 knockdown groups and the control group, since the expression of CD 11b in the knockdown groups was almost similar to that of control group as detected by flow cytometry (Figure 8E).

## Discussion

In recent years, the impact of centrosome abnormalities on human cancers has been attracting attention (Anderhub et al., 2012; Denu et al., 2016; Levine et al., 2017). Centrosomes played vital roles in accurate chromosome segregation during mitosis, making precisely per copy during each cell division and helping to maintain genomic integrity (Neben et al., 2004). Centrosome abnormalities caused spindle formation and dysfunction, chromosome segregation imbalance, triggering subsequent genomic instability and promoting tumorigenesis (Cosenza et al., 2017). Genetic instability was one of the common features of AML. Neben et al. revealed that centrosomal aberrations as a possible cause of aneuploidy in AML and the proportion of cells carrying abnormal centrosomes was associated with increased cytogenetic risk status and poor prognosis (Neben et al., 2004). PLK4 is a regulator of centrosome replication and plays important roles in centriole replication (Maniswami et al., 2018). PLK4 dysregulation led to abnormal number of centrosomes, mitotic defects, chromosomal instability and consequently tumorigenesis. Therefore, inhibition of PLK4 might be a new strategy for the treatment of many cancers, including AML (Zhao and Wang. 2019). The PLK4 high expression always leads to defective cell mitosis, which triggers tumorigenesis (Bettencourt-Dias et al., 2005; Habedanck et al., 2005). Although roles of PLK4 in solid cancers have been studied, there was a controversy in whether PLK4 as an oncogene or tumor suppressor. Our previous RNA-Seq analysis indicated that PLK4 was high expressed in the AML cells, but roles and mechanisms of PLK4 in the leukemiagenesis of AML were still unclear. So, to inhibit the expression of PLK4 either by the PLK4 inhibitor or lentivirus-mediated knockdown in the AML cell lines would help us to clarify roles and mechanisms of PLK4 in the leukemiagenesis of AML, and thus provide clues in whether PLK4 was a potential target for AML clinical treatment and therapy. In this study, lentivirus-mediated PLK4 interference and PLK4 inhibitor Centrinone was used to investigate roles and mechanisms of PLK4 in the pathology of AML. Our results suggested that knockdown the expression of PLK4 inhibited the cell proliferation, colony formation of AML cell lines, promoted the cell apoptosis, and caused the G2/M phase cell cycle arrest of AML cell lines. These observations herein may provide clues in roles of PLK4 in the leukemiagenesis of AML.

Previous studies indicated that PLK4 was high expressed in several human cancers, including hepatocellular carcinoma, colorectal cancer, gastric cancer, glioblastoma, neuroblastoma, breast cancer, and lung cancer (Zhang et al., 2021), and had a close relationship with the progression of cancers. Due to the vital roles of PLK4 in the regulation of centrosome replication and the pathology of cancers, more and more PLK4 inhibitors were developed, such as Centrinone, Centrinone-B, CFI-400495 and YLT-11 (Mason et al., 2014; Wong et al., 2015; Kawakami et al., 2018b; Denu et al., 2018; Lei et al., 2018; Kerschner-Morales et al., 2020; Zhao et al., 2021; Singh et al., 2022). The already known studies information about above PLK4 inhibitors, such as structures, mainly studied cancer cells and effects of these inhibitors on the biological behaviors have been summarized in Table 1. Since the Centrinone was a selective and reversible PLK4 inhibitor with the lowest Ki values among all known inhibitors, in this study the Centrinone was used to treat AML cell lines to explore roles and mechanisms of PLK4 in the pathology of AML. Our results suggested that Centrinone inhibited the proliferation of AML cells in a dose- and time-dependent manner. This observation was consistent with previous studies which indicated that PLK4 promoted the progression of cancers (Zhang et al., 2021). Moreover, our study revealed that Centrinone induced the apoptosis of AML cells in a dose-dependent manner. Mechanismly, Centrinone induced the apoptosis of AML cells *via* increased the expression of cleaved Caspase-3 and cleaved PARP. This result implied that Centrinone induced the apoptosis of AML cells by activating the Caspase signaling pathway.

**TABLE 1:** The summary of the structure, studied cancer cells and roles of the inhibitors of PLK4.

Inhibitors	Structure	Target	Mainly studied cancer cells	Effects	References
Centrinone	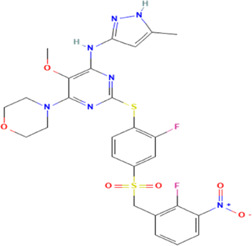	PLK4	Ewing’s sarcoma cells; Cervical carcinoma cells	Cell proliferation inhibition; cell cycle arrest; cell apoptosis induction; colony formation inhibition	Kerschner-Morales et al. (2020); Wong et al. (2015)
Centrinone-B	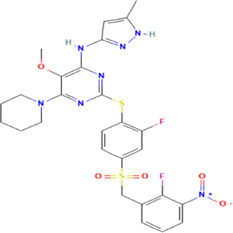	PLK4	Melanoma cells; Prostate cancer cells	Cell proliferation inhibition; cell apoptosis induction; colony formation inhibition	Denu et al. (2018); Singh et al. (2022)
CFI-400495	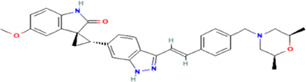	PLK4	Breast cancer cells; Lung cancer cells; Prostate cancer cells; Diffuse large B-cell lymphoma cells	Cell proliferation inhibition; cell cycle arrest; cell apoptosis induction; colony formation inhibition	Mason et al. (2014); Kawakami et al. (2018a); Singh et al. (2022); Zhao et al. (2021)
YLT-11	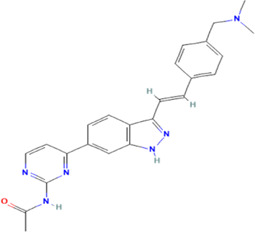	PLK4	Breast cancer cells	Cell proliferation inhibition; colony formation inhibition; cell apoptosis induction	Lei et al. (2018)

The structures of inhibitors were taken from database PubChem.

The cell cycle engine is located downstream of the confluence of oncogenic signaling networks and it is an important target for cancer diagnosis and therapy. The cell cycle dysregulation was responsible for the aberrant cell proliferation of cancer cells (Williams and Stoeber. 2012). Previous studies showed that PLK4 was involved in the regulation of the cell cycle and stress response, and its abnormal expression levels were associated with the progression of tumors (Raab et al., 2021). In this study, our results indicated that knockdown or Centrinone inhibited the expression of PLK4 caused AML cells in the G2/M phase cell cycle arrest by decreasing the expression of Cyclin A2, Cyclin B1, and CDK1. This result was consistent with previous studies which suggested that PLK4 was involved in the regulation of the cell cycle and promoted the development of cancers (Raab et al., 2021).

The leukemia stem cell (LSC) was the source of leukemia relapse and the death of AML patients (Knorr and Goldberg. 2020). In this study, effects of PLK4 on the stemness maintenance of LSC were detected by the colony formation assay. Our results suggested that the colony size and the number of colonies were significantly decreased when the expression of PLK4 was downregulated. Thus, our results suggest that downregulation of PLK4 reduces the stemness maintenance of LSC.

All in all, our results suggested that either the Centrinone or the lentivirus-mediated interference the expression of PLK4 inhibited cell proliferation, induced cell apoptosis and suppressed cell colony formation of AML cells. This study provided an experimental basis for PLK4 in the leukemiagenesis of AML. However, our understanding of PLK4 aberrant expression in cancer development is far from adequate, and more research is needed to investigate the novel mechanisms of their involvement in maintaining genomic stability and new strategies for AML targeted therapies.

## Data Availability

The original contributions presented in the study are included in the article/Supplementary Material, further inquiries can be directed to the corresponding authors.
