# Supplementary material for: Effects of the PLK4 inhibitor Centrinone on the biological behaviors of acute myeloid leukemia cell lines
Source: Front Genet. 2022 Aug 16;13:898474. doi: 10.3389/fgene.2022.898474 (PMC9424683; doi:10.3389/fgene.2022.898474)

Cell: MOLM-13
Drug: Centrinone (0,100,200nM)

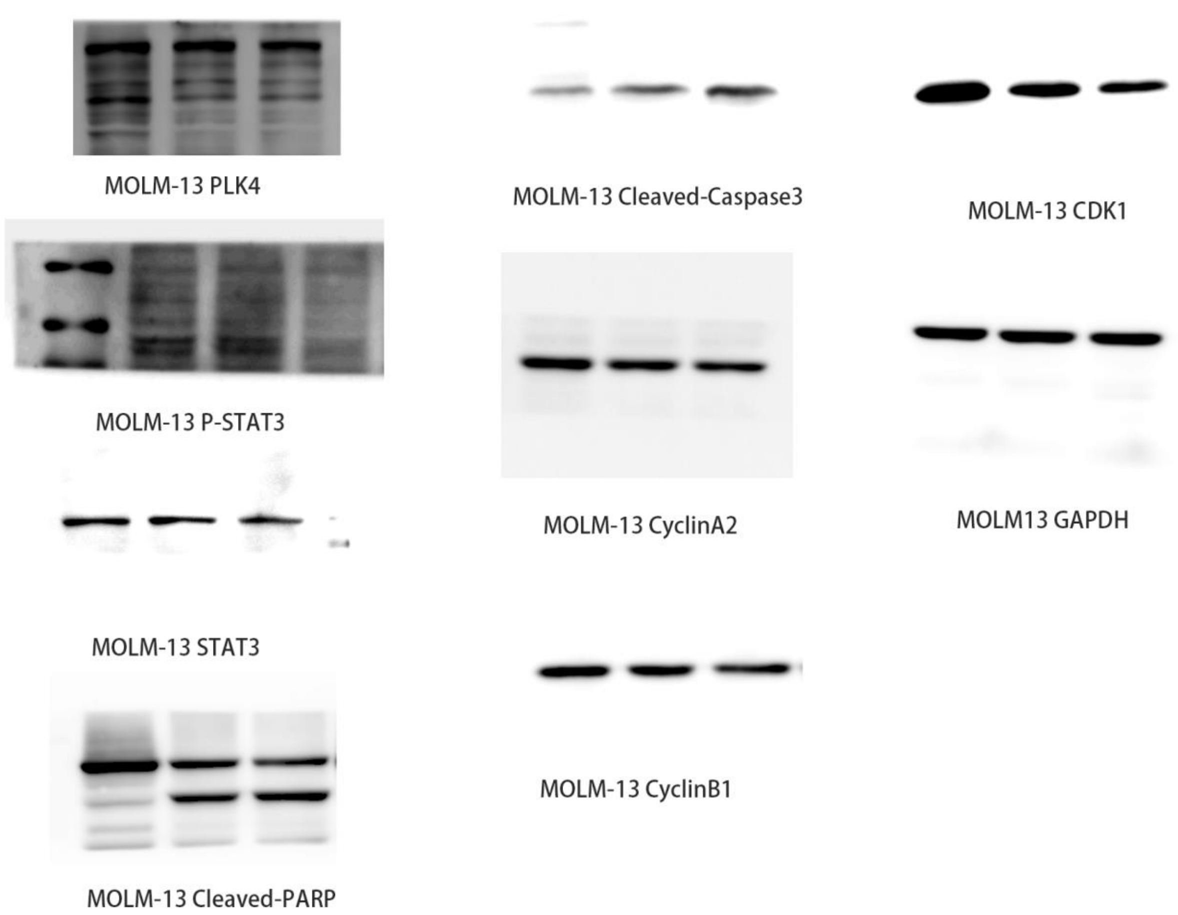

Cell: OCI-AML3
Drug: Centrinone (0,100,200nM)

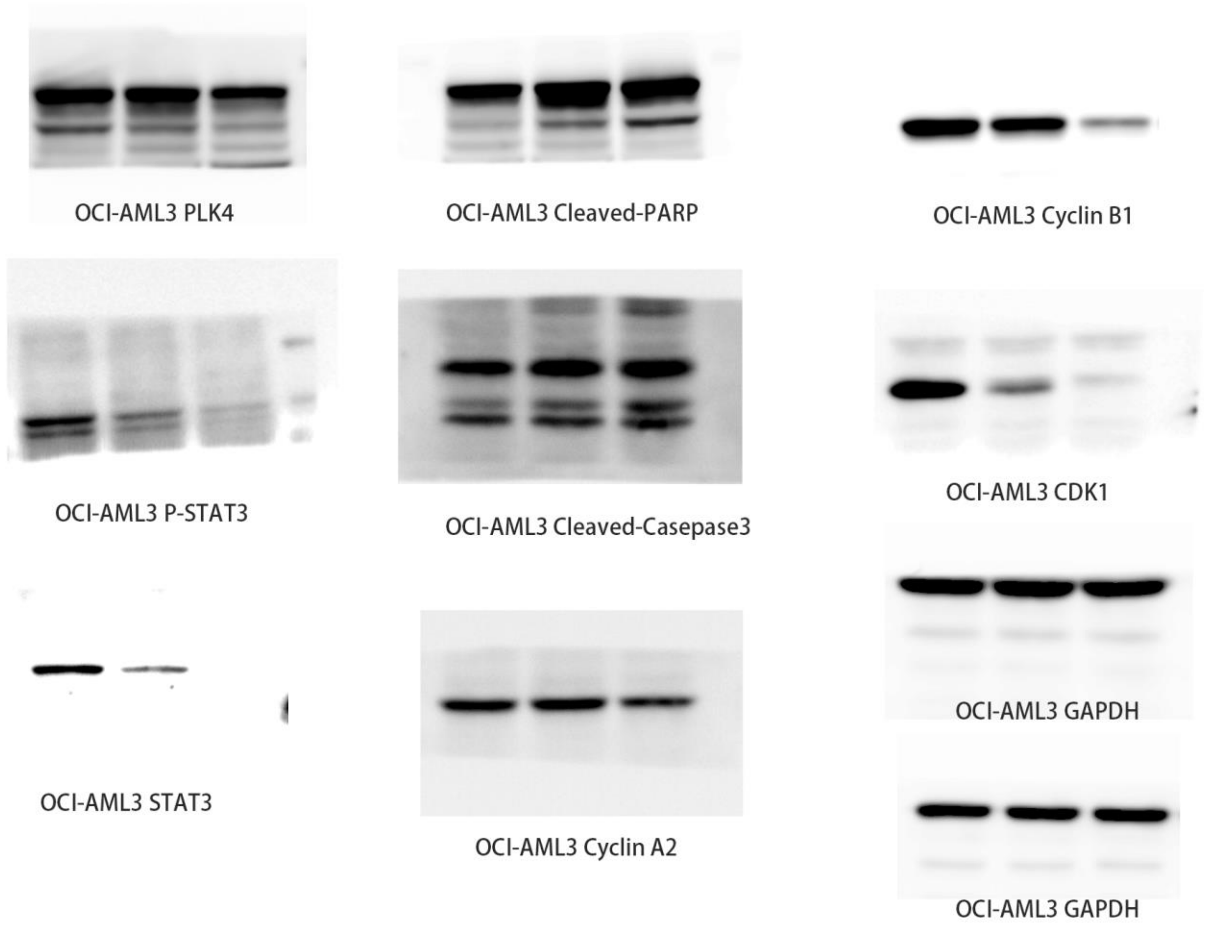

Cell: KG-1
Drug: Centrinone (0,100,200nM)

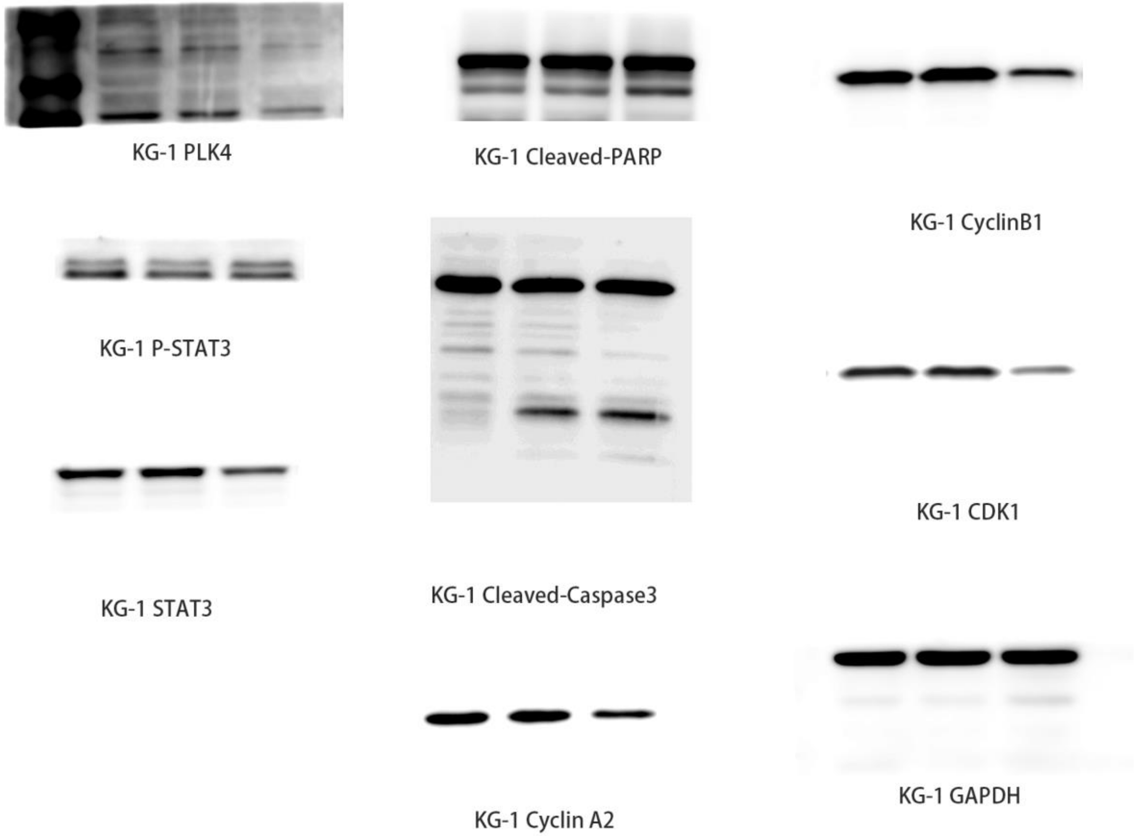

OCI-AML3-sh-EGFP
OCI-AML3-sh-PLK4-1
OCI-AML3-sh-PLK4-2

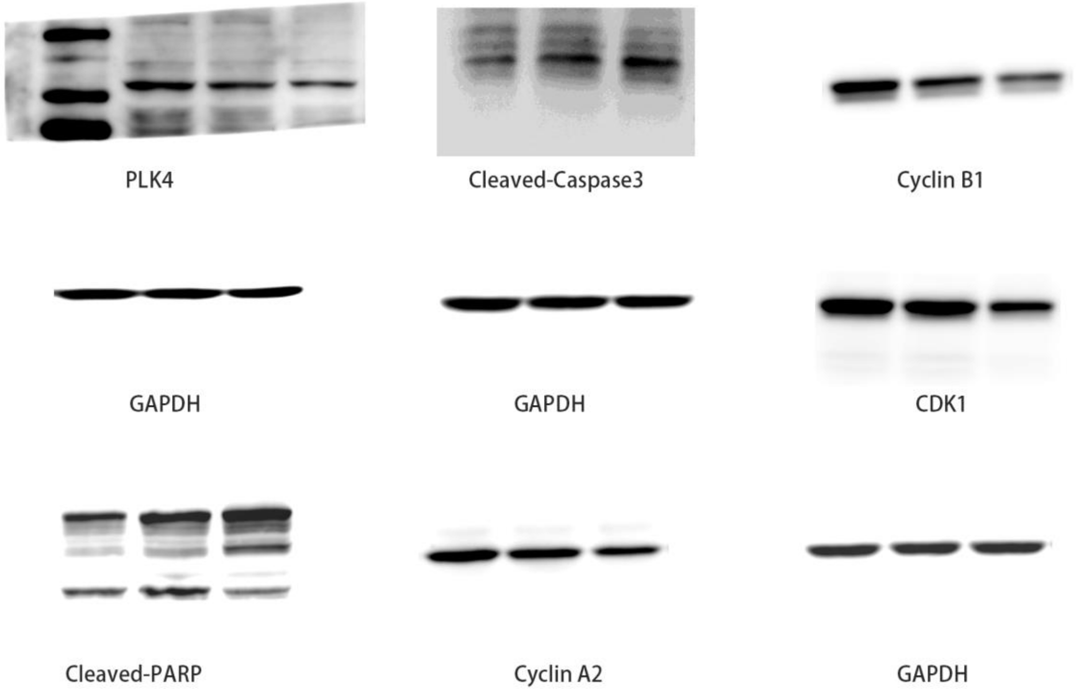

Supplement: Supplementary file 1 [file DataSheet1.PDF]
